# Dysfunctional Cognition and Work-Related Outcomes: A Systematic Literature Review

**DOI:** 10.3390/ejihpe16050069

**Published:** 2026-05-17

**Authors:** Christian Scholtes, Petru Lucian Curșeu, Sabina Ramona Trif

**Affiliations:** 1 Department of Psychology, Babeș-Bolyai University, 400015 Cluj-Napoca, Romania; christianscholtes@psychology.ro (C.S.); sabinatrif@psychology.ro (S.R.T.); 2 Department of Organization, Open Universiteit, 6419 AT Heerlen, The Netherlands

**Keywords:** dysfunctional cognition, early maladaptive schema, burnout, stress, work performance

## Abstract

This systematic literature review synthesizes and integrates research on dysfunctional cognition (DC) in organizational settings, addressing the lack of a coherent model explaining how cognitive vulnerabilities shape work-related outcomes. Following the PRISMA guidelines, we analyzed 41 manuscripts (selected from more than 4523 initial and secondary search hits), leading to an integrative model of DC at work. Results show that DC is activated by job demands, alongside other demanding situational and contextual features. The model specifies maladaptive appraisal processes as the cognitive–affective mechanism through which DC shapes (dis)engagement in the task and relational domains at work and ultimately impacts outcomes such as well-being, stress, burnout, performance, and decision quality. It further incorporates job, personal, and social resources as buffering contingencies that promote adaptive appraisal and attenuate the detrimental role of DC. By integrating insights from clinical, cognitive–behavioral and organizational research, this review advances theory in three ways: (1) by emphasizing the role of appraisal as the cognitive–affective mechanism linking DC to work (dis)engagement, (2) by embedding DC in the job demands–resources model and identifying job demands as activating conditions and resources as regulatory factors for dysfunctional cognitive dynamics at work and (3) by differentiating between task and interpersonal (dis)engagement as domain-specific paths through which self-focused and relational schema impact work-related outcomes. The integrative DC model provides a foundation for future research using longitudinal and mixed-method designs, and for more fine-grained examinations of how specific forms of DC relate to distinct cognitive–affective pathways and work-related outcomes, while offering practical implications for developing schema-informed and resource-based interventions in organizations.

## 1. Introduction

While functional cognition, including bounded rationality (Lieder & Griffiths, 2020), has been the object of interest in numerous organizational studies, dysfunctional cognition (DC) has been much less often researched in work settings. DC is understood to involve irrational beliefs involving absolutistic demands (Lindner et al., 1999), and we define early maladaptive schemas (EMSs) as inflexible, generalized, and persistent cognitive structures (Young et al., 2006). Another perspective is represented by essential, absolute, and sufficient truths, which self-perpetuate by influencing the selection of the information to be processed and by the manifestation of overcompensating behaviors (Young, 1999). EMSs have a self-defeating impact on the individual, yet are difficult to change, as their potential change is perceived as a threat (Young, 1999). They result from unmet needs and dysfunctional interpersonal relations during early childhood, as well as stressful or traumatic events from other contextual experiences (Curșeu et al., 2000; Dickie et al., 2025; J. A. Simpson et al., 2012; Young et al., 2006).

Meta-analytic evidence documented DC as a psychological vulnerability leading to psychological maladjustment (Bishop et al., 2022; Janovsky et al., 2020), yet existing integrative approaches to cognition in organizations (Hodgkinson & Healey, 2008) have largely neglected the role of DC in shaping work-related outcomes. In particular, the literature lacks a systematic understanding of: (1) how DC is activated and regulated in work contexts; (2) how it operates as a cognitive vulnerability that shapes the appraisal process of task and interpersonal cues, and (3) how it impacts task and interpersonal engagement and ultimately work-related outcomes (such as well-being and performance). To address this gap, we set out to review the literature on DC at work and provide a first integrative synthesis of empirical evidence on the role of DC at work, consolidating findings across clinical, organizational, and cognitive–behavioral research traditions. Building on a general model of DC as a behavioral antecedent (Janovsky et al., 2020; Young et al., 2006) and the insights of the job demands–resources model (JD-R, Bakker & Demerouti, 2007), we put forward a contingency model of DC at work that makes three important contributions to the literature: (1) it describes the cognitive–affective mechanisms through which DC influences work strain, (2) it integrates the role of job demands and resources as activating and buffering factors of DC, and (3) it distinguishes the dual impact of DC on task-related and interpersonal functioning at work.

## 2. Theoretical Background

Dysfunctional schemas are latent cognitive structures that remain dormant until triggered by specific life events, circumstances, or stressors (Dozois & Beck, 2008; Scher et al., 2005). Once activated, EMSs influence how an individual receives, codes, stores, and retrieves information (Bishop et al., 2022). They tend to associate with other dysfunctional schemas in “cognitive constellations”, leading to the processing of information congruent with the given schemas, while other information is discarded (Young, 1999). They generate illogical and misguided behaviors, significantly impacting mental health and psychological well-being (Young et al., 2006). As such, DC is a hallmark of cognitive vulnerability, manifested under challenging or stressful personal and professional circumstances, and affects both the individual’s mood as well as information selection and processing.

Cognitive schemas may act as adaptive structures by providing fast and simplified sense-making and interpretation of reality (Gilboa & Marlatte, 2017; Schmidt et al., 1999), as long as they are subject to restructuring when exposed to sufficient information that differs from or contradicts their content (Thornton, 2018). This ability to update schemas in response to external changes is considered a critical sensing capability (Hodgkinson & Healey, 2011; Gamble, 2020). However, when adaptive regulation is missing and the context, including work-related tasks and relations, keeps being misrepresented, these structures of interpretation become dysfunctional cognitive schemas, leading to risky, even harmful, decisions and behaviors (Thornton, 2018).

In order to provide a clearer conceptual organization of DC, we distinguish between self-focused and relational schema (Baldwin, 1992; Cross et al., 2002). They represent cognitive structures that guide information processing, influencing how individuals interpret themselves, their social environment, and the interpersonal interactions they engage in (Andersen & Chen, 2002; Baldwin, 1992). Self-focused schemas are cognitive representations about oneself, including beliefs about competence, vulnerability, or social adequacy (e.g., defectiveness, failure, or unrelenting standards) that shape the way in which personal and interpersonal experiences are perceived and evaluated (Young, 1990). Relational schemas are cognitive representations about others and the interpersonal relationships encompassing beliefs and expectations about how others will behave and one will be treated in social interactions (Baldwin, 1992), including dysfunctional expectations of rejection or mistrust (Young, 1990). The distinction between self and relational cognitive schemas is consistent with research on the relational-interdependent self, showing that individuals differ in the extent to which they define themselves in terms of close relationships and how they tend to experience and make sense of their interpersonal interactions (Cross et al., 2002). Importantly, self-focused and relational schemas are shaped not only by early life experiences but also by ongoing social stressors such as discrimination that can confirm and accentuate dysfunctional relational schemas (Mikrut et al., 2022) and trauma that enhances alienation and contributes to the development of maladaptive relational expectations and self-evaluations (McIlveen et al., 2020). Table 1 presents an overview of self-focused and relational dysfunctional cognitive schema with illustrative examples from the reviewed organizational literature.

One of the first studies to distinguish the mechanisms and impact of DC in both non-work and work-related circumstances was provided by Judge and Locke (1993). The study aimed to better understand both subjective well-being and job satisfaction by adopting perspectives based on the cognitive theory of depression, focusing on irrational, dysfunctional, repetitive, and automatized thought processes (Beck, 2002), akin to EMSs. The results showed that the presence of dysfunctional thought processes leads to both increased likelihood of unhappiness and job-related dysfunctional thought processes (such as unreasonable performance standards), and that the latter significantly affects job satisfaction (Judge & Locke, 1993).

More recent studies on the presence of DC in work settings have shown its impact to be significant—it diminishes decision comprehensiveness (Scholtes et al., 2024), it leads to lack of confidence in self and in others, which in turn puts collaboration and mutual support under strain (Lindner et al., 1999), it increases the probability of rumination, which then leads to increased isolation and withdrawal from helping behaviors, such as knowledge sharing (Wu et al., 2022), and it enables relationship conflict and counterproductive work behaviors (Muntean et al., 2025). While not directly related to work contexts, irrational beliefs, such as the need to be successful or to be liked at all costs, or the need for absolute comfort or for absolute fairness (Lindner et al., 1999), once activated, generate a self-depreciative confirmatory bias and also a pessimistic expectation about future events (Bruch et al., 1989), shaping, in a work setting, the appraisal of one’s own capabilities and of tasks and interactions.

Given the relative gap in the literature on DC in organizations, an aspect which has been virtually neglected by previous approaches that discussed cognition in organizations (Hodgkinson & Healey, 2008), and through further building on previous models of DC at work (Judge & Locke, 1993), we aim to put forward an integrative model in which contextual factors at work activate DC that then triggers downstream influences through cognitive and emotional appraisals, therefore impacting task-related and interpersonal behaviors in work settings. We set out to answer two key research questions: (1) How does DC impact organizational behavior and work-related outcomes? (2) Which are the contingencies that shape the impact of DC on organizational behavior and work-related outcomes, through accentuation or attenuation?

## 3. Materials and Methods

The present review focused on the impact of DC in organizations, alongside contingency factors that shape its impact. Although the present systematic review effort has not been pre-registered, we closely followed the PRISMA guidelines regarding the manuscript search, selection, and analysis (Page et al., 2021).

**Figure 1:**
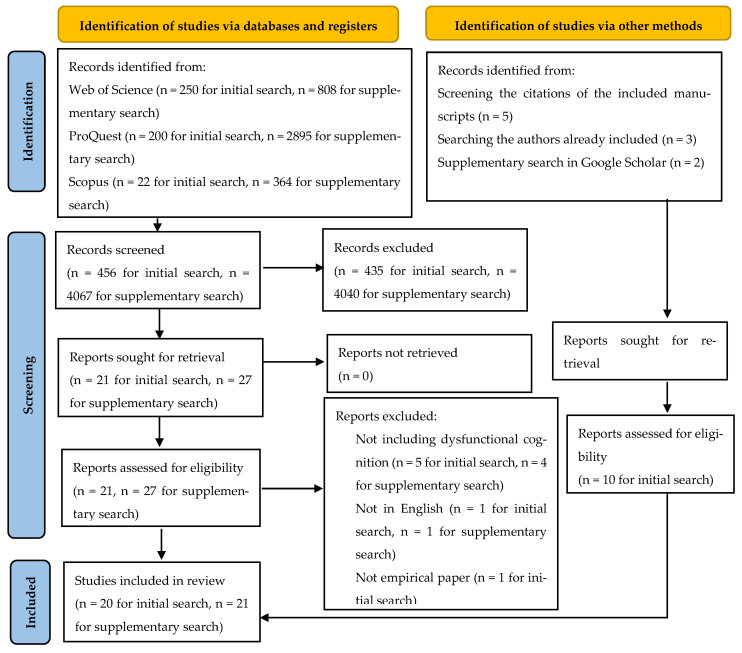
PRISMA flow diagram. Source: (Page et al., 2021).

For the first search, we used the following search query: (“dysfunctional cognition” OR “early maladaptive schem*” OR “dysfunctional cognitive schem*”) AND (organization* OR work OR employee* OR team* OR leader* OR manager*) NOT disorder*. No additional filters or search limits were used. Search results from all databases were exported and merged, duplicates were removed before analysis, and the reference lists of the eligible studies were additionally examined to identify other relevant studies to be included in the review. The initial search produced a total of 472 manuscripts. After excluding duplicates, 456 manuscripts were included in the first selection process. The criteria used for the selection were: the manuscripts must be empirical papers, in English, with dysfunctional cognition as a variable, and set in an organizational context. Two independent research assistants selected the manuscripts based on title and abstract, with a third researcher making decisions in cases of disagreement. After the initial step, 21 manuscripts were selected for full-text review. The same initial independent research assistants reviewed the 21 manuscripts, with a third researcher solving any disagreements. Based on the full-text review, 10 papers were selected to be included in the review. To expand the sample of manuscripts, we reviewed the reference list of the papers included in the review, adding 5 new manuscripts. We also searched Google Scholar for the authors of the 15 manuscripts and included 3 new ones. Finally, we did a supplementary check on Google Scholar, leading to 2 new manuscripts. Thus, the initial sample consisted of 20 manuscripts (see Figure 1 for the PRISMA diagram).

Upon feedback received from the reviewers, a second supplementary search was initiated in the same databases (i.e., Web of Science, ProQuest, Scopus) using an expanded search query: (“dysfunctional cognition” OR “early maladaptive schem*” OR “dysfunctional cognitive schem*” OR “cognitive distortion*” OR “maladaptive belief*” OR “irrational belief*” OR “dysfunctional attitude*” OR “maladaptive coping” OR “maladaptive schema*”) AND (organization* OR work OR employee* OR team* OR leader* OR manager*) NOT disorder*). As the search resulted in a considerable number of manuscripts, we employed a series of filters and limitations regarding language (English), manuscript type (article, peer-reviewed), and, in the case of Web of Science only, only specific research areas were selected, such as psychology, business economics, public environmental occupational health, social sciences other topics, or public administration. The second search produced a total of 4067 results. The criteria used for the selection were consistent with those employed for the initial search. After the title and abstract-based selection, we removed any overlapping studies that had already been included based on the initial search. A total of 27 new manuscripts were included in the full-text selection. Based on the full-text review, 21 new papers were selected to be further included in the review. In total, 41 cumulative, non-redundant manuscripts were included in the review.

Two independent research assistants extracted (a) the paper, (b) the variables included, (c) the research design, (d) the number of participants and their profiles, and (e) the results of the data analysis (the results are summarized in the table presented in Appendix A). In order to assess the risk of bias for the studies included in the systematic review, we adapted the Joanna Briggs Institute (JBI) Critical Appraisal Checklist for Analytical Cross-Sectional Studies (Moola et al., 2020). As the index is constructed for medical studies, one element was considered not applicable to our study (i.e., measuring exposure). The criterion related to measurement of the condition was adapted into “measurement of the main variable”, the one related to dysfunctional cognition. Furthermore, the one related to the outcome measurement was adapted into “measurement of other variables included in the study”. We retained the criteria related to sample inclusion, the detailed description of the subjects, the identification of confounding factors, the presence of strategies for dealing with confounding factors, and the appropriateness of statistical analyses. We considered that confounding variables were identified even if they were just discussed in the Limitations Section. The JBI checklist clearly distinguishes between identifying possible confounding factors and employing strategies for dealing with them. The criterion related to identification states that measurement could be an important part of identification, but it may be difficult, especially related to behaviors, attitudes, or lifestyle-related variables (Moola et al., 2020). Given that the subject of the present systematic literature review includes such variables, their recognition and discussion in the Limitations Section was considered enough to check the identification of confounding variables. We used the criterion related to dealing with confounds to assess the extent to which there was statistical control of potential confounding factors.

Two independent researchers completed the assessment, with discrepancies solved through discussion. A total of 31 studies reported information regarding the inclusion criteria in the sample, while 9 did not, but all studies (41) offered information regarding the participants. Regarding the measurement of the main variable, 32 papers presented valid and reliable measurements, while 9 studies were marked as “unclear” due to not providing or providing very limited information regarding the reliability and validity of the scales used. Regarding the measurement of predictors and/or criteria, 33 presented valid and reliable measurements, while 8 studies were marked as “unclear” due to reasons described above. Regarding confounding factors, 19 studies identified confounding factors, even if just in the Discussion Section, while 22 did not address this issue. Only 9 papers presented strategies for dealing with confounding variables by statistical control, while 32 did not propose any control strategies. All papers were evaluated as using the appropriate statistical analysis. The strongest points of the papers involve the statistical analysis and the description of the sample. The main limitations involve identifying confounding variables and dealing with them.

We proceed by summarizing the results in line with our research questions and then we provide an integrative theoretical model building on key concepts and methods from clinical (Janovsky et al., 2020), cognitive–behavioral (Young et al., 2006) and organizational (Bakker & Demerouti, 2007; Jovanović et al., 2025) research traditions. This systematic literature review was not pre-registered.

## 4. Results

Before presenting the summary of our findings concerning the relation between DC and various work-related outcomes, we will reflect on how DC were operationalized, evaluated, and which research designs were used across the included studies. The vast majority of studies operationalized DC as early maladaptive schemas (EMSs), maladaptive coping modes, and irrational beliefs, and used validated self-report assessment methods. Most commonly, early maladaptive schemas were evaluated using different versions of the Young Schema Questionnaire, while irrational beliefs and cognitive distortions were evaluated using domain-specific self-report scales adapted to work contexts. All studies relied on self-report measures, and we could not identify studies that used implicit cognitive measures, behavioral observations or other in-depth evaluations of DC. This pattern of results suggests that the current literature on DC at work primarily captures explicit, conscious cognition, with little to no attention for implicit or automatic information processing.

In terms of research designs, most of the studies were cross-sectional, with only a few adopting cross-lagged designs. Given the predominance of cross-sectional evidence, we refrain from making definitive claims regarding the antecedents and consequences of DC in work contexts. However, consistent with generic theoretical models of dysfunctional cognition (Beck, 2002; Dozois & Beck, 2008), the reviewed studies allow for a distinction between conditions under which DC is more likely to be activated and the work-related outcomes with which it is associated. A small number of studies (Čopková, 2024; Selçuk & Grassie, 2023) examined the association between DC and dark triad traits, suggesting that maladaptive cognitive patterns co-occur with socially aversive personality characteristics. A recurring finding is that stressful work conditions activate DC-related vulnerabilities, particularly with respect to burnout (especially emotional exhaustion) and strain-related symptoms. Although the samples varied in terms of occupational background, in high-demand occupational contexts (e.g., healthcare, teaching, psychological services), EMSs involving rigid standards, punitive self-evaluation, subjugation, interpersonal mistrust, and self-sacrifice are frequently implicated as predictors of emotional exhaustion and general distress (Abeltina & Rascevska, 2021; Bamber & McMahon, 2008; Bay & Novinrouz, 2022).

Overall, the studies show that DC (EMSs, maladaptive coping modes, and irrational beliefs) is associated with burnout-related outcomes, psychological distress, and dysfunctional organizational behavior. With only one notable exception (Gkontelos et al., 2023), DC had detrimental effects for work-related outcomes, either directly or moderated by various contextual variables. In terms of self-focused schemas (defectiveness, failure, self-worth, perfectionism, low frustration tolerance) studies have shown a strong association with reduced well-being at work and maladaptive psychological outcomes such as stress and burnout (Falco et al., 2017; Judge & Locke, 1993; Houghton & Jinkerson, 2007; Santarpia et al., 2023). In terms of relational schema (mistrust, rejection, approval seeking or subjugation), studies documented clear effects on interpersonal relationship outcomes and counterproductive work behaviors, such as conflict and bullying (Curșeu et al., 2025; Goh & Oei, 1999; Guppy & Weatherstone, 1997; Jang & Lee, 2022; Mohammadi et al., 2020; Muntean et al., 2025; Robertson & Dunsmuir, 2013; Yildirim et al., 2015). Mixed or integrative schemas and coping modes (general dysfunctional modes such as global irrational beliefs, dysfunctional coping modes, general cognitive distortions) (El-Shokheby, 2020; Eyni et al., 2025; Houghton & Jinkerson, 2007; S. Simpson et al., 2019) were important antecedents of stress, burnout, anxiety, emotional exhaustion, and reduced well-being at work. The table presented in Appendix A summarizes the key findings of the 41 studies included in the review with the studies revealing mixed or contextual detrimental effects of DC being marked in bold. In the following section we will summarize the main findings alongside the RQs that guided our SLR.

### 4.1. Burnout and Distress

Across multiple occupational groups (health workers, teachers, dentists, psychologists, therapists, trainees, emergency psychologists), DC, especially EMSs and maladaptive schemas and coping modes, is linked to a higher likelihood of burnout (Abeltina & Rascevska, 2021; Balevre, 2001; Bay & Novinrouz, 2022; Huk et al., 2019; Kaeding et al., 2017; Fitzhardinge et al., 2025; Mironova & Kovalevskaia, 2024), particularly emotional exhaustion (EE; Chriqui et al., 2025; Khorshidian et al., 2017; S. Simpson et al., 2019; Smout et al., 2022). DC is therefore a vulnerability factor associated with higher probabilities of burnout (especially EE) and sometimes with depersonalization and reduced personal accomplishment/performance (Bamber & McMahon, 2008). DC is also an important antecedent of work-related stress (Bernard, 2016; El-Shokheby, 2020; Tan, 2004; Turner et al., 2022), triggering dysfunctional appraisal processes (Guppy & Weatherstone, 1997). Certain schemas or coping modes appear as central cognitive network elements or bridge nodes in the associative burnout network (e.g., punitiveness, unrelenting standards, subjugation, dependence/incompetence; modes like demanding parent, detached protector, angry child, self-aggrandizer; Abeltina & Rascevska, 2021).

Moreover, some EMSs show clear specificity across occupational groups, reflecting selection and attrition processes most likely driven by person–environment fit mechanisms (Schneider, 1987). For example, compared to mental health professionals, other health professionals present higher levels of abandonment and defectiveness schemas. In the specific case of mental health professionals, years of experience are positively related to the vulnerability to harm or illness cognitive schema (Dang et al., 2019).

Several studies (Curșeu et al., 2025; Eyni et al., 2025; Khorshidian et al., 2017; S. Popov et al., 2015; Turner et al., 2022) link DC to depression, anxiety, psychiatric symptoms, and general distress. DC is therefore also associated with clinically relevant psychological symptoms, often via cognitive–affective mechanisms such as rumination tendencies and negative affect (Curșeu et al., 2025).

One vignette experimental study in organizational settings tested emotional mechanisms that explain the association between DC and work strain. In particular, irrational beliefs are accompanied by maladaptive emotions (anxiety, rage, guilt), while rational beliefs are associated with adaptive emotional reactions such as annoyance, sadness, or regret as functional signals (Spörrle et al., 2006). Such adaptive emotions were generally judged as more functional, implying downstream implications for interpersonal effectiveness and emotionally intelligent behavior (Spörrle et al., 2006). Empirical evidence shows that DC shapes emotional quality and emotional reactivity functionality, which plausibly cascades into workplace behavior (communication, conflict handling, etc.) that ultimately impacts well-being and adaptation at work.

### 4.2. Counterproductive Work Behaviors and Dysfunctional Organizational Processes

Beyond burnout and EE, DC shows meaningful links to workplace conflict, psychological safety, and counterproductive work behaviors (Muntean et al., 2025), with clear implications for higher-level decision process outcomes (Scholtes et al., 2024). DC is a key antecedent of dysfunctional interpersonal behaviors, including workplace bullying and relationship conflict (Jang & Lee, 2022; Muntean et al., 2025), while teachers’ irrational beliefs predicted negative relational climates in classrooms (Robertson & Dunsmuir, 2013). Moreover, DC was shown to negatively impact the quality of clinical judgments during psychotherapeutic interventions (Saddichha et al., 2012), while managerial rationality fosters decision comprehensiveness only when managers score low rather than high on DC (Scholtes et al., 2024). Unrealistic performance standards and perfectionistic irrational cognition predict workaholism as a compensatory mechanism for the fear of failure, ultimately leading to exhaustion and negative work–life balance (Akutsu et al., 2022; van Wijhe et al., 2013; Falco et al., 2017). A distinctive result is presented in Gkontelos et al. (2023), reporting a positive association between the irrational beliefs of demands for justice and innovative work behaviors in teachers. Such results may reflect a paradoxical positive outcome of DC in work contexts, possibly rooted in a dual regulatory focus of approach–avoidance that is an essential driver for creativity and innovation (Oțoiu et al., 2025).

Early evidence on dysfunctional thought processes at work (Judge & Locke, 1993) shows that individuals who engage more in dysfunctional thinking are also more likely to report unhappiness. Consistent with an emotion-focused appraisal mechanism, subjective well-being then predicts job satisfaction. Judge and Locke (1993) have differentiated between dysfunctional cognition in general and job-related dysfunctional thought processes, showing that job-related dysfunctional processes reduce job satisfaction and subsequently trigger job-avoidance cognitions (withdrawal, avoidance). Based on these results, we can conclude that DC shapes appraisal tendencies at work in two ways, namely by affecting task engagement and by shaping engagement in interpersonal interactions at work.

### 4.3. Contingencies of DC Outcomes: Personal and Job-Related Social Resources

The magnitude as well as the direction of the association between DC and work-related outcomes were moderated by contextual and situational variables. Empirical evidence shows that DC effects are contingent upon the availability of social resources and psychosocial safety (Curșeu et al., 2025; Muntean et al., 2025), and the availability of individual protective resources such as resilience and hardiness (Bay & Novinrouz, 2022; Smout et al., 2022).

A distinction between personal resources and job-related social resources can be made based on the clustering of results (Tesi et al., 2025). On one hand, personal resources such as hardiness and resilience attenuate the association between DC and the likelihood of burnout (Bay & Novinrouz, 2022; Smout et al., 2022). Therefore, these personal resources reduce the likelihood of dysfunctional appraisal modes and act as a buffer for the pathways between DC and burnout and work strain. Such factors appear to reduce the relationship between maladaptive cognitions and EE, consistent with models of psychological capital and adaptive coping (Qiu et al., 2025). In occupational groups exposed to chronic demands, resilient individuals may engage in more effective regulation strategies and reduce the deleterious effects of maladaptive cognitive–affective spirals associated with schemas and irrational beliefs (Smout et al., 2022). Taken together, evidence supports the conceptualization of resilience and hardiness as individual-level attenuating contingencies, moderating the degree to which DC is translated into strain-related outcomes.

Studies also indicate that job-related social resources (e.g., peer support, social acceptance at work) diminish the extent to which maladaptive cognitions translate into repetitive negative thinking and internalizing symptoms, respectively, consistently attenuating the harmful downstream impact of DC (Curșeu et al., 2025; Muntean et al., 2025). In particular, the role of social resources appears to be robust for schemas involving interpersonal threat (e.g., mistrust) by weakening the positive association between DC and rumination (Curșeu et al., 2025). In organizational contexts where DC is involved in conflict processes, social support and psychologically safe environments appear to function as protective contextual conditions, limiting escalation into counterproductive behavior (Muntean et al., 2025). Thus, social support emerges as a key buffering contingency, reducing the strength of DC-related effects on both well-being and maladaptive workplace behavior.

An important insight of our analyses is the relative distinctiveness of the adaptive and maladaptive cognitive structures. Houghton and Jinkerson (2007) report that constructive cognitive strategies alleviate dysfunctional thought processes, yet this association was rather weak. Moreover, Turner et al. (2022) reported two distinct cognitive profiles, one described by low irrational beliefs and elevated self-determination and one described by elevated irrational beliefs and low self-determination. On the one hand, such results reveal the potentially protective role of constructive cognitive strategies and open avenues for cognitive restructuring workplace interventions. On the other hand, they emphasize the role of work contingencies in activating either functional or dysfunctional thought processes. In what follows, we further integrate and elaborate the mechanisms and contingencies through which DC is associated with work-related outcomes, pointing out relevant research directions.

## 5. Discussion and Future Research Directions

We have synthesized in a descriptive and evidence-based manner empirical findings on the main patterns of association between DC, contextual contingencies and various work-related outcomes. In what follows, we move beyond descriptive synthesis and we build on established theoretical models from clinical and cognitive psychology (Andersen & Chen, 2002; Baldwin, 1992; Beck, 2002; Bruch et al., 1989; Dozois & Beck, 2008; Scher et al., 2005; Schmidt et al., 1999; Young et al., 2006) to organize the empirical evidence in a process-oriented and integrative model of DC at work. The model specifies the contextual conditions that activate DC in work settings, the cognitive–affective appraisal mechanisms through which DC shapes task-related and interpersonal functioning at work as well as the role of personal, job-related and social resources as buffering contingencies that attenuate the maladaptive downstream consequences of DC activation. In doing so, we intend to advance a theoretically grounded synthesis of the mechanisms and contingencies linking DC to work-related outcomes and to point out relevant research directions.

### 5.1. An Integrative Model of DC in Organizations

We argued that exacerbated work/job demands as well as stressful work contexts are likely activating conditions for DC (Abeltina & Rascevska, 2021; Bamber & McMahon, 2008; Bay & Novinrouz, 2022; Curșeu et al., 2025; Jovanović et al., 2025; S. Popov et al., 2015). In addition, several studies emphasized intermediary psychological and interpersonal processes (rumination, negative affectivity, conflict), which function as pathways through which DC becomes behaviorally consequential (Curșeu et al., 2025; Goh & Oei, 1999; Muntean et al., 2025).

Empirical evidence shows that irrational beliefs are related to occupational strain by shaping maladaptive appraisal processes, whereby existing stressors are interpreted in absolutist or catastrophic terms, thereby increasing the likelihood of adverse stress reactions (Curșeu et al., 2025; S. Popov et al., 2015). Collectively, these findings support the interpretation of job demands as an activating context for DC, increasing their downstream impact on both well-being and work functioning. This evidence supports the interpretation of DC as a context-sensitive predictor of task-related as well as interpersonal outcomes at work, with implications for both occupational interventions (e.g., stress reduction, psychological safety initiatives) and individual-focused approaches (e.g., schema-informed interventions, resilience-building, cognitive restructuring).

DC is theorized to influence stress/strain outcomes partly through biased appraisal, such as catastrophizing, rigid demands, heightened threat sensitivity, and punitive self-evaluation (Lindner et al., 1999). Nikolova et al. (2021) provide empirical support for the role of ruminative tendencies as proximal cognitive mechanisms linking situational threat appraisal to work-related strain outcomes. Building on the transactional model of stress and coping (Lazarus & Folkman, 1984), the authors conceptualize rumination as sustained repetitive cognition triggered by appraisals of uncontrollable and overwhelming situational demands (Nikolova et al., 2021). In this context, rumination is a key appraisal mechanism through which DC can translate into strain and work disengagement (Curșeu et al., 2025). The JD-R model also states that job, personal, and social resources (including supportive peer interactions, social acceptance, and psychologically safe climates) can reshape the DC activated by job demands (Bakker et al., 2023) by reducing the perceived intensity of stressors and increasing perceived coping capacity (Bakker & Demerouti, 2007). We contend that they do so by activating adaptive cognitive and emotional situational appraisals, consistent with stress and coping models in which resources reduce strain not only by lowering demands but also by reshaping appraisal processes and promoting adaptive coping responses (Goh & Oei, 1999).

In other words, job demands trigger maladaptive appraisal reflected in threat-amplifying cognitive interpretations (e.g., catastrophizing, rumination, self- and other-blame) and disproportionate and prolonged negative affect, leading to task and relational disengagement. Job, personal, and social resources, on the other hand, trigger more constructive cognitive interpretations and support effective emotional regulation, therefore attenuating the tendency to engage in maladaptive appraisal. In our integrative model, cognitive–affective appraisal processes link the activation of DC to task and interpersonal engagement at work.

Taken together, the results of our SLR show that DC is a cognitive vulnerability whose impact on organizational behavior and work-related outcomes is shaped by: (1) job demands and stressors that activate and amplify maladaptive appraisals, (2) individual psychological resources that support adaptive appraisal and coping strategies, and (3) job-related social resources that buffer distress pathways and protect interpersonal functioning. The observed mediators, such as rumination, catastrophizing, negative affectivity, self- and other-blame indicate that DC influences work outcomes primarily by shaping cognitive–affective processing and interpersonal behavior under stress. Given their deeply ingrained natures, DC can be considered a stable antecedent of primary appraisal processes (Goh & Oei, 1999; Guppy & Weatherstone, 1997).

Our model fills a gap in the literature on cognition in organizations by addressing the dysfunctional nature of cognition and cognitive processes, aspects that were virtually neglected by previous integrative approaches (Hodgkinson & Healey, 2008). Although DC originates in early childhood experiences (Young et al., 2006), several contextual variables at work can activate EMSs and activate cognitive and emotional appraisal processes. Previous research has documented the effect of DC on dysfunctional interpersonal behaviors (Janovsky et al., 2020) as well as dysfunctional work-related behaviors (Bamber, 2006; Muntean et al., 2025). Therefore, it is our contention that task-related and interpersonal behaviors at work are impacted by the cognitive and emotional appraisals activated by DC.

**Figure 2:**
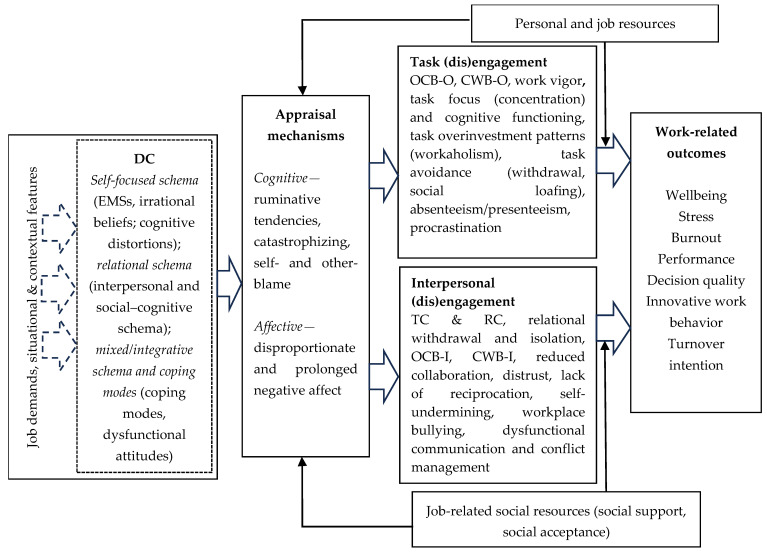
An integrative model of dysfunctional cognition at work. Note: OCB-O = organizational citizenship behavior related to the organization, OCB-I = organizational citizenship behavior related to individuals, CWB-O = counterproductive work behavior related to the organization, CWB-I = counterproductive work behavior related to individuals; TC = task conflict; RC = relationship conflict.

Given the distinction between the different types of DC identified in this review and presented in Table 1, we argue that self-focused dysfunctional schemas tend to activate appraisal modes with a dominant impact on task-related functioning and (dis)engagement and ultimately impact well-being at work and individual performance (individual work-related outcomes). Relational dysfunctional schemas are expected to impact more broadly on interpersonal functioning and (dis)engagement at work and trigger a negative relational climate that ultimately reduces team and organizational performance (collective work-related outcomes). Finally, the mixed/integrative schemas and coping modes, due to their global nature and their affective evaluative dimension, are expected to have a more pervasive and global impact on task-related as well as relational functioning at work, having the most detrimental influence on both individual as well as collective work-related outcomes. Although we do not hypothesize exclusive influences on task-related and interpersonal interactions at work, we do expect a certain domain-specific influence of different types of DC identified in this review that call for multilevel explorations of the impact DC has on individual and collective work-related outcomes. The overall integrative model of how DC impacts work-related outcomes is presented in Figure 2.

### 5.2. Future Research Directions

Building on the integrative model and the gaps identified in the literature, we will further present a few research directions. We first integrate in Table 2 the main outcomes summarized across different types of DC identified in this review, and we then discuss several research directions informed by the integrative framework presented earlier.

A first direction for further exploration is how job demands function as activating contingencies for DC at work. Most of the research to date in the context of the JDR model has considered job demands and resources as moderators in the relation between task and interpersonal engagement and work-related outcomes (Bakker & Demerouti, 2007; Jovanović et al., 2025; Fernández-Salinero et al., 2026; Tesi et al., 2025). Nevertheless, job demands can also be related to the activation of DC. For example, workload perceived as excessive or unfair can be associated with schemas of mistrust, dependence, or failure (S. Simpson et al., 2019). In line with proposition eight stated in the revised JDR theory (Bakker et al., 2023), job demands can activate maladaptive cognitions, which in turn foster self-undermining behaviors. Furthermore, when individuals with DC also experience lack of resources due to the novelty of the job demands, such as in job transitions or during major change processes, we expect that the damaging impact on interpersonal engagement and work-related outcomes will be amplified. Therefore, we call for future research to investigate the triggering role of job demands for DC in work settings.

Job-related social resources, on the other hand, can serve a protective role and social support, for example, by inducing a more functional appraisal of the work conditions under high distrust schema (Muntean et al., 2025). Given the traumatic etiology of EMSs (Curșeu et al., 2000; Dickie et al., 2025; J. A. Simpson et al., 2012; Young et al., 2006), we assume that, when individuals with latent DC will experience a competitive work environment, with high job demands and lack of collaboration or support among peers, the likelihood of distress will increase significantly, up to potentially experiencing the deterioration of general well-being and mental health. Moreover, personal resources such as resilience and optimism can impact the appraisal tendencies and reduce the deleterious effects of DC. Future research could therefore investigate the role of personal, social and job resources in shaping the appraisal tendencies triggered by DC.

Little emphasis was put on the relation between DC and coping styles at work; future research could explore the dynamics between DC and different coping styles (functional vs. dysfunctional) in work contexts. It may be worthwhile to explore the degree to which individuals carrying DC from early childhood, by being involved in therapeutic or developmental processes, become aware of their dysfunctional thoughts and interpretations, and shift their responses to stressful work situations from dysfunctional to functional coping styles. Moreover, in the reviewed studies, little emphasis is placed on how the work environment can shape the emergence of DC. Some studies have explored the role of dark triad personality dimensions (Machiavellianism, narcissism, psychopathy) as related to DC (Čopková, 2024; Selçuk & Grassie, 2023), yet the causal role of work experiences was not explicitly addressed.

As people spend a considerable amount of time at work, and work experiences can also include traumatic, discriminatory, or even harassing interactions with the potential to generate DC (Mikrut et al., 2022; McIlveen et al., 2020), future research could also explore the role of dysfunctional work environments as potential triggers of DC. A particular line of research in this area concerns leader–follower interactions, especially in the context of toxic leadership practices, as such leadership can spur negative and persistent relational spirals that can foster the emergence of DC. Recent dyadic evidence indicates that leaders and followers mutually influence each other’s work states and behavioral intentions, thereby positioning the leader–follower dyad as a critical relational unity for understanding the interpersonal diffusion of strain processes (Caniëls & Curseu, 2024a, 2024b). In particular, negative events at work as contextual factors triggering DC can generate EE for both leaders and their followers, consistent with a negativity spiral. In contrast, positive work events increase work engagement and job satisfaction (Caniëls & Curseu, 2024b), and dyadic leader–follower exchanges can activate protective relational resource transfer and foster resilient behaviors (Caniëls & Curseu, 2024a). We call for multilevel studies that focus on the relational dynamics in teams and leader–member dyads as regulatory systems shaping the translation of DC into work-related outcomes by impacting cognitive–affective appraisal tendencies as well as interpersonal regulatory processes and the interpersonal resource exchange.

Rather than being confined to private or work settings, DCs are cognitive vulnerabilities that are activated under challenging, demanding, or stressful conditions such as role conflict. Building on the differentiation–integration framework advanced in recent research on hybrid work (Manole et al., 2025), future studies could examine how evolving work arrangements and broader societal and technological transitions reconfigure the activation and regulation of DC across interrelated work domains. Increased integration provided by flexible work for various personal roles could reduce the salience of DC and alleviate the detrimental effect of role conflict as an activating factor for DC at work and at home. The reduced integration at the team level that is triggered by flexible work arrangements could, however, accentuate the effects of DC at the team level (assuming that team contexts activate DC in their members during interactions). For example, elevated DC at the team level may impact how team members engage in knowledge sharing or knowledge withholding, and virtual communication may enhance the likelihood that interpersonal cues are misinterpreted through the activation of DC. Future research could explore communication behavior in teams in face-to-face and virtual settings, as it is driven by activated DC.

Beyond documenting such direct associations between DC and work outcomes, this review advances theory by developing a contingency-based framework that clarifies when and how DC is linked to work-related outcomes. We call for a better integration of theoretical models stemming from clinical psychology (Janovsky et al., 2020) and cognitive–behavioral models (Young et al., 2006) with models that traditionally explain well-being and performance at work. Our integrative model shows that, next to job demands and resources, and next to work-related attitudes and behavioral intentions, DCs are cognitive vulnerabilities that shape task and interpersonal engagement at work. Insights from clinical psychology on the relations between DC and general behavioral tendencies can be extrapolated to better understand dysfunctional behaviors at work.

## 6. Practical Implications

At a practical level, developmental programs in organizations seem to be built on the implicit assumption that participants are operating quasi-exclusively with rational cognition and that what is missing for them to interact and perform better is concrete knowledge on how to better tackle work-related challenges. Generally, coaching seems to be the sole developmental approach wherein dysfunctional cognition is acknowledged, typically under the label of limiting beliefs about self and others. We would therefore recommend that organizational practitioners make space also for the topic of dysfunctional cognition when designing developmental programs, by addressing it as a latent cognitive vulnerability, and by making explicit that it can affect employees both personally (leading potentially to burnout and emotional exhaustion) and interpersonally (leading to conflictual, harmed relationships) and that, mirroring its negative outcomes, it can be mitigated by building personal (resilience and hardiness) and interpersonal (social support and psychological safety) resources. In addition, we would recommend embedding individual coaching conversations in higher-stakes development programs, as it provides, akin to the schema therapy (Young et al., 2006), a structured reflection environment where dysfunctional scripts and beliefs can be brought to awareness, can be explored, and can potentially undergo a restructuring process.

## 7. Limitations

Our systematic literature review has several limitations. First, our review reveals the absolute dominance of cross-sectional designs in the exploration of DC at work, a fact that provides a rather static perspective on DC at work and limits causal inferences, underscoring the need for experimental, longitudinal, and diary-based designs capable of capturing the dynamic activation process and the reciprocal effects between DC, contextual conditions, and work-related outcomes. The findings reported in our review are therefore vulnerable to retrospective bias, lack causal tractability, and may reflect inflated associative relations.

Second, the studies included in this review reflect significant heterogeneity concerning the operationalization of DC, including EMSs, maladaptive coping styles, and irrational beliefs. Although these constructs share significant similarities in terms of theoretical grounding, their assessment and theoretical mechanisms differ. Such conceptual and methodological diversity limits direct comparability across studies and inferences related to the activation and regulation mechanisms at play for different types of DC.

Third, the empirical evidence includes various occupational groups, yet the focus on healthcare professionals, teachers, and psychologists limits the generalizability of our inferences. These professions can be seen as intensive in emotional labor and stress, with high levels of social interaction that may gravely impact others, such as students or patients (K. Chen & Chen, 2026; J. Chen & Ren, 2026; Clarke et al., 2023; Feng et al., 2024; Kariou et al., 2021; Sun et al., 2025), offering a fertile ground for the emergence and manifestation of DC, especially in the case of social schemas. Workplaces with more limited human contact and lower interdependence levels may present different result patterns regarding social schemas. Moreover, the sample is geographically skewed towards European and Middle Eastern samples with a clear focus on individual outcomes, with relatively little attention to team or organizational level outcomes. Differences in cultural orientations may also limit the extent to which the results can be generalized. For example, a meta-analysis shows that, in collectivistic countries, the differentiation between depressed and healthy individuals based on cognitive vulnerabilities is lower (Bartucz et al., 2022).

Fourth, relatively few studies explicitly tested moderation and mediation models, limiting the grounding of the contingencies presented in our theoretical model. Additionally, despite following the PRISMA guidelines and using different search strategies, the results reported in this paper may be subject to publication and language bias as non-English papers and practitioner-oriented reports have been omitted.

Finally, the present review has not been pre-registered on any platform. This reduces the transparency of the process, limiting the credibility of the methodology used, as the protocol had not been peer-reviewed before the data collection and analysis began. Moreover, it raises the possibility of effort duplication, as other researchers could not be informed of the present effort (Pennington, 2025). Yet, the review authors prepared a review protocol from the beginning, including the search string, the databases for the search, the inclusion and exclusion criteria, and the analysis strategy.

## 8. Conclusions

This systematic literature review presents the first integrative synthesis of empirical research on DC in organizational settings. Building on theoretical insights from clinical (Janovsky et al., 2020), cognitive–behavioral (Young et al., 2006), and organizational traditions (Bakker & Demerouti, 2007; Jovanović et al., 2025), we put forward an integrative model that DCs (EMSs, maladaptive coping modes, irrational beliefs) are markers of cognitive vulnerability that activate cognitive and emotional appraisal tendencies that shape the way in which employees engage with tasks and the interpersonal relations at work, ultimately impacting their work-related outcomes. Across occupational contexts, DCs as cognitive vulnerabilities are associated with heightened stress, emotional exhaustion, burnout symptoms and dysfunctional organizational behaviors. Our model indicates that DCs impact work engagement and work-related outcomes through cognitive–affective mechanisms, such as ruminative tendencies and negative affect that explain the translation of situational demands at work into adverse work-related outcomes. This review positions DCs as a central, yet underexplored determinant of employee well-being and organizational functioning. By specifying the cognitive and affective mechanisms and contingencies through which DCs operate in work settings, the study provides a foundation for more nuanced theory development calling for methodologically rigorous research and evidence-based interventions aimed at fostering healthier and model-adaptive work environments.

## Figures and Tables

**Table 1:** Types of self-focused and relational dysfunctional schema.

Category	Type of Cognition	Definition	Example in Reviewed Studies
Self-focused schemas	Early maladaptive schemas (self-related)	Stable negative beliefs about the self, including inadequacy, failure, or vulnerability	Defectiveness, failure, dependence, unrelenting standards
	Irrational beliefs (self-evaluative)	Rigid, absolutist beliefs about personal performance or worth	“I must always succeed”, low frustration tolerance
	Cognitive distortions (self-related)	Biased interpretations of personal experiences	Catastrophizing, overgeneralization
Relational schemas	Interpersonal/relational schemas	Cognitive representations of others and social interactions, including expectations about treatment by others	Mistrust, rejection, abandonment, demand for fairness
	Social–cognitive schemas (contextual)	Broader expectations about the social world shaped by experiences such as discrimination or stress associated with perceptions of disconnection from others and/or the self	Social vigilance, invalidation concerns, abandonment, social alienation, social inadequacy
Mixed/integrative schema and coping modes	Schema modes/cognitive–affective patterns	Dynamic thinking patterns combining self and relational schemas with emotional appraisal and behavioral tendencies	Detached protector, demanding parent, dysfunctional attitudes

**Table 2:** Types of self-focused and relational dysfunctional schema.

Cognition Type	Main Outcomes Studied	No. of Studies	Illustrative Studies	Future Research Directions
Self-focused schemas	Burnout, stress, impaired well-being at work	~12–15	Falco et al. (2017); Santarpia et al. (2023); Judge and Locke (1993); S. Simpson et al. (2019)	Examine activation under job demands; explore longitudinal effects and individual coping/resource mechanisms; test effectiveness of cognitive restructuring interventions in the work context
Relational schemas	Dysfunctional interpersonal outcomes, conflict, bullying	~6–8	Curșeu et al. (2025); Jang and Lee (2022); Yildirim et al. (2015); Guppy and Weatherstone (1997); Muntean et al. (2025)	Investigate team- and dyad-level dynamics (e.g., leader–follower) and outcomes in multilevel studies; explore the role of social resources and rational beliefs as moderators
General dysfunctional cognitions	Broad outcomes (performance, stress, dysfunctional behavior)	~10–12	El-Shokheby (2020); Eyni et al. (2025); Houghton and Jinkerson (2007); Turner et al. (2022); S. Popov et al. (2015)	Develop multi-method approaches (beyond self-report); test causal mechanisms and test the effectiveness of cognitive restructuring strategies

## Data Availability

The data involved in this study are fully presented in the paper.

## Abbreviations

The following abbreviations are used in this manuscript:

EMS	Early Maladaptive Schema
DC	Dysfunctional Cognition
JDR	Job Demands–Resources Model
EE	Emotional Exhaustion

## Appendix A

**Table A1:** Summary of studies included in the review.

No	Paper	Variables Included	Research Design	Participants (Number and Profile)	Results
1	Abeltina and Rascevska (2021)	Burnout, early maladaptive schemas, and schema modes	Cross-sectional	562 participants from Latvia, with an average age of 41 (SD = 11.82), 82% female	Several early maladaptive schemas (punitiveness, unrelenting standards, subjugation, dependence, and incompetence) and several schema modes (demanding parent, detached protector, angry child, and self-aggrandizer) are significantly related to burnout.
2	Bamber and McMahon (2008)	Early maladaptive schemas, profession, occupational stress	Cross-sectional	243 participants from UK, with an average age of 42, 72% female, 50.2% working in mental health	EMSs correlate positively with emotional exhaustion and detachment, while most EMSs correlate negatively with personal accomplishment. Enmeshment, self-sacrifice, and insufficient self-control are significantly and positively related to the total number of days of sick leave.
3	Bay and Novinrouz (2022)	Early maladaptive schemas, hardiness, and burnout	Cross-Sectional	142 participants from Iran, first-grade elementary school teachers. The average working background was 16.62 years, aged between 34 and 32	Impaired performance and autonomy, impaired limits, and hypervigilance/inhibition had a significant positive correlation with burnout. Hardiness had a significant negative correlation with burnout.
4	Chriqui et al. (2025)	Early maladaptive schemas and professional burnout	Cross-sectional	61 participants from Morocco, practicing dentists, with an average age of 33.13, 32 women, 61.3% single	Significant positive relationships were reported between vulnerability impairment, entitlement, dependency, insufficient self-control, mistrust, and high-performance expectation and burnout.
5	Curșeu et al. (2025)	COVID-19 rumination, mistrust cognitive schemas, depression, anxiety, social acceptance, peer support	Cross-sectional	1010 participants from Romania, with an average age of 42.04, 938 women	There was a significant positive association between mistrust and anxiety and depression, partially mediated by COVID-19 rumination. Social acceptance attenuated the association between mistrust and COVID-19 rumination while peer support attenuated the association between COVID-19 rumination and depression and anxiety.
6	Dang et al. (2019)	Early maladaptive schemas, health profession (mental health or other health profession)	Cross-sectional	128 participants from India, 64 were mental health professionals	Other health professionals presented higher levels of abandonment and defectiveness schemas compared to mental health professionals. Males presented higher levels of abandonment, mistrust, entitlement/superiority, admiration/recognition seeking, and emotional inhibition compared to women.
7	**Goh and Oei (1999)**	Dysfunctional attitudes, stress, primary appraisal, secondary appraisal, coping strategies	Cross-sectional	244 undergraduates from Australia, with an average age of 22.6 years (SD = 7.6), 164 women, students with at least six months of work experience	In the high-occupational-stress model, dysfunctional attitudes affected primary appraisal and were affected by stress outcomes. In the low-occupational-stress model, dysfunctional attitudes had no significant relationship with any stress variables.
8	Kaeding et al. (2017)	Early maladaptive schemas, burnout, physical health symptoms	Cross-sectional	1172 participants from multiple countries (US, Australia, Canada, UK), average age of 28.42 (SD, 82.3% women = 6.29)	The high-burnout group presented significantly higher levels of early maladaptive schemas. Unrelenting standards was the only early maladaptive schema that could correctly classify trainees who experienced high burnout. Dependence, unrelenting standards, social isolation, and insufficient self-control together led to the highest percentage of correct classifications. The high-burnout group experienced significantly more physical health symptoms than the low-burnout group.
9	Khorshidian et al. (2017)	Early maladaptive schemas, burnout, mental disorder symptoms	Cross-sectional	276 participants from Iran, 46.6% women, average age of 34.4 (SD = 7.88), average work experience 11 years (SD = 7.95)	Significant positive correlations were reported between all mental disorder symptoms and all early maladaptive schema burnout symptoms. EMSs were positively and significantly correlated with emotional exhaustion and decline in performance. Regression analyses revealed that aggression predicted all three burnout dimensions, and paranoid ideation predicted decline in performance. Based on regression analyses, impaired autonomy/performance predicted emotional exhaustion and decline in performance. Vigilance/inhibition predicted decline in performance.
10	Fitzhardinge et al. (2025)	Adverse childhood experiences, emotional exhaustion, defectiveness/shame, unrelenting standards, self-sacrifice and subjugation schemas	Cross-sectional	383 therapists from multiple countries, with an average age of 46 years (SD = 12.79), 89% women	There was a significant positive correlation between adverse childhood experiences and emotional exhaustion. Unrelenting standards and subjugation as early maladaptive schemas mediated the effect of adverse childhood experiences and emotional exhaustion.
11	Mironova and Kovalevskaia (2024)	Early maladaptive cognitive schemas, professional burnout	Cross-sectional	36 participants from Russia, with an average age of 36 years old, 86.1% female, with an average work experience of 11 years	There were significant positive correlations between self-sacrifice, unrelenting standards, negativity/pessimism, punitiveness, and approval seeking/recognition-seeking and burnout. 13 out of 18 early maladaptive schemas presented significant positive correlation with at least one burnout dimension, especially those from the other-directedness, and hypervigilance and inhibition domains.
12	Muntean et al. (2025)	Dysfunctional cognition, team conflict, psychological safety, counterproductive work behaviors, social support	Cross-sectional	3689 teachers from Romania, average age of 42.99 (SD = 9.64), 3328 women from 331 schools	Dysfunctional cognition had a significant positive association with task conflict, relationship conflict, and counterproductive work behaviors. Dysfunctional cognition had a significant negative association with psychological safety. Within-schools, task conflict, relationship conflict, and psychological safety mediated the effect of dysfunctional cognition on counterproductive work behaviors while between-schools, task conflict acted as a significant mediator. Social support acted as a buffer against the negative effects of dysfunctional cognition, with a significant within-school interaction effect for task conflict, relationship conflict, and psychological safety.
13	B. Popov and Popov (2013)	Irrational and rational beliefs, cognitive appraisal, occupational stress, and stressful working conditions	Cross-sectional	221 participants from Serbia, with an average age of 39.2 years (SD = 12.1), 57.5% women, with an average work experience of 15.6 years (SD = 11.5)	In the first set of analyses, stressors and job insecurity positively and significantly predicted distress, and adding coping strategies (problem solving, seeking social support, avoidance) significantly improved the model. The same effects were present for burnout, with a smaller contribution on the part of coping strategies. When introducing rational and irrational beliefs, stressors, rational, and irrational beliefs predicted distress, with no interaction effect present. Stressors and irrational beliefs had a significant positive effect on burnout, with no interaction effect and no effect on the part of rational beliefs.
14	S. Popov et al. (2015)	Irrational beliefs, stressors, stress	Cross-sectional	186 teachers from Serbia, with an average age of 40.2 (SD = 10.1), 75% women	There was a significant effect of stressors on general stress. Adding irrational beliefs as a mediator reduced the impact of stressors, but the relationship remained significant. Irrational beliefs partially mediated the relationship between stressors and general stress.
15	B. Popov et al. (2016)	Negative affect, irrational beliefs, negative experiences at work, psychosomatic complaints, absenteeism	Cross-sectional	499 employees from 18 Serbian organizations, with an average age of 39.5 (SD = 10.45), 69.5% women	Negative affectivity partially mediated the effect of negative experiences at work and psychosomatic complaints. Psychosomatic complaints fully mediated the relationships between negative experiences at work, irrational beliefs, their interaction, and negative affectivity and absenteeism. Out of the psychosomatic complaints, physical symptoms and fatigue significantly and directly impact absenteeism.
16	Saddichha et al. (2012)	Maladaptive cognitive schemas and biases	Cross-sectional	100 mental health professionals from India, with an average age of 24.9 (SD = 3.9), 51% women, with an average of work experience of 2.8 years (SD = 2)	Psychiatrists and other mental health professionals show higher levels of social isolation, enmeshment, and insufficient self-control schemas compared with psychologists. Psychiatrists show higher levels of unrelenting standards schema than other mental health professionals and psychologists. Age presents significant positive correlations with self-sacrifice and unrelenting standards schemas and significant negative correlations with abandonment schema.
17	**Scholtes et al. (2024)**	Managerial rationality, decision comprehensiveness, and dysfunctional cognitive schemas	Cross-sectional	270 managers from Romania, with an average age of 40.72 years (SD = 7.82), 145 women, with an average work experience of 17.68 years (SD = 7.62)	Managerial rationality and managerial dysfunctional cognitive schemas do not have a significant direct effect on decision comprehensiveness, yet there is a significant interaction effect, with high levels of dysfunctional cognitive schemas reversing the effect of managerial rationality on decision comprehensiveness.
18	S. Simpson et al. (2019)	Early maladaptive cognitive schemas, maladaptive coping modes, burnout	Cross-sectional	443 English-speaking international psychologists, 80.4% women, 52.8% married, 51.9% from Australia	All EMSs and maladaptive coping modes were significantly positively correlated with emotional exhaustion (highest correlations for the disconnection and rejection domains). The highest correlations regarding the maladaptive coping modes were for the detached protector. Regression analyses revealed that emotional exhaustion was predicted by job demands, early maladaptive cognitive schemas (specifically emotional inhibition, abandonment, and mistrust/abuse), and maladaptive coping modes (specifically detached protector).
19	Smout et al. (2022)	Resilience, maladaptive coping, job demands, emotional exhaustion	Cross-sectional	425 English-speaking international psychologists, with average age of 42.79 (SD = 11.55), 80.7% women, 53% married, 52% from Australia	Each coping mode was a significant predictor for emotional exhaustion (detached protector and bully as the strongest predictors) and the interaction between coping modes and resilience, was significant, for compliant surrender as a coping mode. Each coping mode (separately) significantly mediated the relationship between job demands and emotional exhaustion.
20	**Spörrle et al. (2006)**	Adaptive emotions, maladaptive emotions, rational thinking, irrational thinking, functionality, emotional states perception and modification, social identity, satisfaction with organizations	Vignette experiment	113 participants, with an average age of 31.2 years, 80 women, 49% students	In the vignettes in which the person was described in rational terms, participants attributed adaptive emotions. In the vignettes in which the person was described in irrational terms, participants attributed maladaptive emotions. In most cases (with one exception), adaptive emotions were rated as more functional compared to the maladaptive ones. In all cases, the rational person is assumed to perceive and modify their emotional states more compared to the irrational one. Identification with the rational person significantly and positively correlated with satisfaction with the organization. Identification with the irrational person significantly and negatively correlated with satisfaction with the organization.
21	**Akutsu et al. (2022)**	Competitive work environment, workaholism, cognitive distortions, subjective unhealthiness	Cross-sectional	9716 employees from Japan, 51% male, mean age 46, from a wide range of industries and educational backgrounds	Competitive work environment was positively related to subjective unhealthiness, partly through workaholism. Cognitive distortions strengthened the positive association between competitive work environment and both workaholism and subjective unhealthiness and amplified the indirect effect via workaholism.
22	Balevre (2001)	Irrational thinking patterns, burnout thoughts, burnout behaviors	Cross-sectional	192 practicing nurses from a large urban hospital in the southern U.S.; majority female, mostly White, many under 40	Mistrust, perfection/control, and entitlement-related irrational patterns were positively associated with burnout, especially burnout thoughts. Critical-care nurses showed higher responses on mistrust and entitlement-related patterns than non-critical-care nurses.
23	Bernard (2016)	Teacher irrational beliefs, teacher stress	Cross-sectional, two-study design	Study 1: 792 teachers from Australia; Study 2: 140 employed teachers and 26 stress-retired teachers	Irrational beliefs were positively associated with teacher stress. Self-downing, authoritarianism, demands for justice, and low frustration tolerance were differentially related to specific stressors, and stress-retired teachers reported higher self-downing and low frustration tolerance than employed teachers.
24	Čopková (2024)	Work-related irrational beliefs, dark triad traits	Cross-sectional	355 employees from Slovakia, mean age 36.57, 52.1% female, employed in private and public sectors	Machiavellianism and narcissism positively predicted overall work-related irrational beliefs. Machiavellianism predicted performance demands and failure beliefs, narcissism predicted co-workers’ approval beliefs, and psychopathy predicted control beliefs; some effects differed by gender and sector.
25	El-Shokheby (2020)	Cognitive distortions, academic stress	Cross-sectional	120 female participants from Saudi Arabia, including 63 trainee students and 57 intermediate school teachers	Cognitive distortions were strongly and positively correlated with academic stress and all its dimensions, indicating that higher cognitive distortions were associated with higher levels of academic stress.
26	Eyni et al. (2025)	Social competence, behavioral emotion regulation, cognitive distortions, health anxiety	Cross-sectional	250 psychiatric nurses from Iran, mean age 32.25, 54% female	Social competence, behavioral emotion regulation, and cognitive distortions had significant direct effects on health anxiety. Cognitive distortions also mediated the associations of social competence and behavioral emotion regulation with health anxiety.
27	**Falco et al. (2017)**	Perfectionism, irrational beliefs at work, workaholism, anxiety, depressive symptoms, burnout, negative affectivity	Cross-sectional	770 Italian workers from multiple organizations and one private metal engineering company	Self-oriented and socially prescribed perfectionism were positively associated with irrational beliefs at work. Failure beliefs were positively associated with workaholism, and failure partly mediated the relationship between perfectionism and workaholism.
28	**Gkontelos et al. (2023)**	Self-efficacy, irrational beliefs, burnout, innovative work behavior	Cross-sectional	964 primary school teachers from Greece, 77% female, mean age 41.35	Self-efficacy was positively related to innovative work behavior and negatively related to burnout and irrational beliefs. Irrational beliefs were positively associated with burnout and showed a significant indirect role in the relationship between self-efficacy and innovative work behavior. Demands for justice were positively correlated with innovative work behaviors.
29	Guppy and Weatherstone (1997)	Dysfunctional attitudes, coping strategies, psychological well-being, depression	Cross-sectional	274 white-collar public sector employees from Wales, mean age 33.3, 69% female	Dysfunctional attitudes were positively associated with avoidant coping, depression, and poorer general mental health. Problem-focused coping was negatively associated with vulnerable attitudes, while avoidant coping and imperatives predicted poorer mental health outcomes.
30	**Houghton and Jinkerson (2007)**	Constructive thought strategies, dysfunctional thought processes, subjective well-being, job satisfaction	Cross-sectional	269 full-time employees from a medium-sized private university in the United States	Dysfunctional thought processes were negatively associated with subjective well-being and job satisfaction. Their effects on job satisfaction were partially mediated by subjective well-being, while constructive thought strategies showed indirect effects through dysfunctional thoughts and well-being.
31	Huk et al. (2019)	School demands, school resources, teacher irrational beliefs, burnout, self-efficacy	Cross-sectional	77 teachers from the United States, with varied teaching roles and average teaching experience of 12.86 years	Teacher irrational beliefs were positively associated with burnout, emotional exhaustion, and depersonalization. Irrational beliefs predicted burnout above and beyond school characteristics, but did not significantly moderate the relationship between school demands and burnout.
32	Jang and Lee (2022)	Pathological narcissism, interpersonal cognitive distortions, positive organizational culture, workplace bullying	Cross-sectional	236 nurses from South Korea, mean age 32.56, 91.5% female	Workplace bullying was positively associated with pathological narcissism and interpersonal cognitive distortions. In regression analyses, pathological narcissism and interpersonal cognitive distortions positively predicted workplace bullying, whereas positive organizational culture was negatively associated with bullying.
33	**Judge and Locke (1993)**	Dysfunctional thought processes, job dysfunctional thought processes, subjective well-being, job satisfaction, job avoidance	Cross-sectional	217 clerical employees from a university in the United States, mean age 37.7, 87% female	Dysfunctional job thought processes were negatively associated with job satisfaction. Dysfunctional thought processes also had indirect negative effects on job satisfaction via subjective well-being and indirect positive effects on job avoidance through reduced job satisfaction.
34	Mohammadi et al. (2020)	Communication skills, early maladaptive schemas	Cross-sectional	146 pediatric nurses from Iran, mean age 33.66, work experience 1–28 years	Communication skills were negatively associated with several maladaptive schema domains, including impaired autonomy/performance, impaired limits, other-directedness, and disconnection/rejection. No significant association was found with overvigilance/inhibition.
35	Robertson and Dunsmuir (2013)	Teacher irrational beliefs, self-efficacy, pupil behavior, teacher stress	Cross-sectional	58 teachers from five schools in the UK, mean age 35.03	Irrational beliefs were the strongest predictor of teacher stress, with self-efficacy making an additional significant contribution.
36	**Santarpia et al. (2023)**	Irrational beliefs at work, performance expectations fulfillment, well-being at work	Cross-sectional	3576 employees from Italy, 55% male	Irrational beliefs were generally negatively associated with performance expectations fulfillment, which in turn was positively associated with well-being at work. Awfulizing showed negative associations with both performance expectations fulfillment and well-being, while performance expectations fulfillment partially mediated several of these relationships.
37	**Selçuk and Grassie (2023)**	Early maladaptive schemas, dark triad traits, personality, values, preventive/risky behaviors	Cross-sectional	294 hospital workers from Turkey, including health and non-health workers	Sixteen of eighteen early maladaptive schemas predicted lower preventive and higher risky behaviors. Machiavellianism, psychopathy, emotional deprivation, and self-sacrifice acted as mediators linking psychosocial factors to preventive/socialization behaviors.
38	Tan (2004)	Irrational beliefs, job stress, sources of stress, burnout	Cross-sectional	37 occupational therapists from Singapore, mean age 30, 78.4% female	Lower frustration tolerance was associated with greater stress related to patient contact. Irrational self-worth beliefs were associated with stress related to professional value and rewards/recognition.
39	**Turner et al. (2022)**	Irrational beliefs, motivation regulation, persistence, well-being, work engagement, stress, anxiety, depression, anger, procrastination, intention to quit, absenteeism, presenteeism	Cross-sectional, two-study design	Study 1: 362 UK workers; Study 2: 362 UK workers across a broad range of occupations	Workers with profiles characterized by higher irrational beliefs and lower self-determination reported lower life satisfaction, higher depression, anxiety, and stress, more procrastination and intention to quit, and poorer work-related functioning than workers with lower irrational beliefs and higher self-determination.
40	**van Wijhe et al. (2013)**	Work-related irrational beliefs, workaholism, negative affect	Cross-sectional	913 employees from the Netherlands, mean age 43.8, employed across multiple sectors	Performance demands and failure beliefs were positively associated with workaholism, whereas co-workers’ approval and control beliefs were not. These associations remained significant after controlling for negative affect.
41	Yildirim et al. (2015)	Interpersonal cognitive distortions, conflict management styles	Cross-sectional	68 school managers from Turkey, predominantly male, with substantial professional experience	Interpersonal cognitive distortions were positively associated with obliging, dominating, and avoiding conflict management styles. Avoidance of intimacy was negatively associated with integrating conflict management.

Note: references marked in bold reveal mixed or conditional effects of DC on work-related outcomes.

## Appendix B

**Table A2:** PRISMA checklist for the systematic literature review.

Section and Topic	Item #	Checklist Item	Location Where Item Is Reported
TITLE			
Title	1	Identify the report as a systematic review.	Title
ABSTRACT			
Abstract	2	See the PRISMA 2020 for Abstracts checklist.	Abstract
INTRODUCTION			
Rationale	3	Describe the rationale for the review in the context of existing knowledge.	Introduction
Objectives	4	Provide an explicit statement of the objective(s) or question(s) the review addresses.	Introduction
METHODS			
Eligibility criteria	5	Specify the inclusion and exclusion criteria for the review and how studies were grouped for the syntheses.	Methods
Information sources	6	Specify all databases, registers, websites, organizations, reference lists and other sources searched or consulted to identify studies. Specify the date when each source was last searched or consulted.	Methods
Search strategy	7	Present the full search strategies for all databases, registers and websites, including any filters and limits used.	Methods
Selection process	8	Specify the methods used to decide whether a study met the inclusion criteria of the review, including how many reviewers screened each record and each report retrieved, whether they worked independently, and if applicable, details of automation tools used in the process.	Methods
Data collection process	9	Specify the methods used to collect data from reports, including how many reviewers collected data from each report, whether they worked independently, any processes for obtaining or confirming data from study investigators, and if applicable, details of automation tools used in the process.	Methods
Data items	10a	List and define all outcomes for which data were sought. Specify whether all results that were compatible with each outcome domain in each study were sought (e.g., for all measures, time points, analyses), and if not, the methods used to decide which results to collect.	Methods
	10b	List and define all other variables for which data were sought (e.g., participant and intervention characteristics, funding sources). Describe any assumptions made about any missing or unclear information.	Not applicable
Study risk of bias assessment	11	Specify the methods used to assess risk of bias in the included studies, including details of the tool(s) used, how many reviewers assessed each study and whether they worked independently, and if applicable, details of automation tools used in the process.	Methods
Effect measures	12	Specify for each outcome the effect measure(s) (e.g., risk ratio, mean difference) used in the synthesis or presentation of results.	Not applicable
Synthesis methods	13a	Describe the processes used to decide which studies were eligible for each synthesis (e.g., tabulating the study intervention characteristics and comparing against the planned groups for each synthesis (item #5)).	Methods
	13b	Describe any methods required to prepare the data for presentation or synthesis, such as handling of missing summary statistics, or data conversions.	Not applicable
	13c	Describe any methods used to tabulate or visually display results of individual studies and syntheses.	Methods and Appendix
	13d	Describe any methods used to synthesize results and provide a rationale for the choice(s). If meta-analysis was performed, describe the model(s), method(s) to identify the presence and extent of statistical heterogeneity, and software package(s) used.	Discussion
	13e	Describe any methods used to explore possible causes of heterogeneity among study results (e.g., subgroup analysis, meta-regression).	Discussion
	13f	Describe any sensitivity analyses conducted to assess robustness of the synthesized results.	Not applicable
Reporting bias assessment	14	Describe any methods used to assess risk of bias due to missing results in a synthesis (arising from reporting biases).	Not applicable
Certainty assessment	15	Describe any methods used to assess certainty (or confidence) in the body of evidence for an outcome.	Not applicable
RESULTS			
Study selection	16a	Describe the results of the search and selection process, from the number of records identified in the search to the number of studies included in the review, ideally using a flow diagram.	Methods
	16b	Cite studies that might appear to meet the inclusion criteria, but which were excluded, and explain why they were excluded.	Not applicable
Study characteristics	17	Cite each included study and present its characteristics.	Appendix A
Risk of bias in studies	18	Present assessments of risk of bias for each included study.	Methods
Results of individual studies	19	For all outcomes, present, for each study: (a) summary statistics for each group (where appropriate) and (b) an effect estimate and its precision (e.g., confidence/credible interval), ideally using structured tables or plots.	Not applicable
Results of syntheses	20a	For each synthesis, briefly summarize the characteristics and risk of bias among contributing studies.	Limitations
	20b	Present results of all statistical syntheses conducted. If meta-analysis was done, present for each the summary estimate and its precision (e.g., confidence/credible interval) and measures of statistical heterogeneity. If comparing groups, describe the direction of the effect.	Not applicable
	20c	Present results of all investigations of possible causes of heterogeneity among study results.	Results and Discussion
	20d	Present results of all sensitivity analyses conducted to assess the robustness of the synthesized results.	Not applicable
Reporting biases	21	Present assessments of risk of bias due to missing results (arising from reporting biases) for each synthesis assessed.	Not applicable
Certainty of evidence	22	Present assessments of certainty (or confidence) in the body of evidence for each outcome assessed.	Not applicable
DISCUSSION			
Discussion	23a	Provide a general interpretation of the results in the context of other evidence.	Discussion
	23b	Discuss any limitations of the evidence included in the review.	Limitations
	23c	Discuss any limitations of the review processes used.	Limitations
	23d	Discuss implications of the results for practice, policy, and future research.	Discussion
OTHER INFORMATION			
Registration and protocol	24a	Provide registration information for the review, including register name and registration number, or state that the review was not registered.	Review not registered
	24b	Indicate where the review protocol can be accessed, or state that a protocol was not prepared.	Review not registered
	24c	Describe and explain any amendments to information provided at registration or in the protocol.	Review not registered
Support	25	Describe sources of financial or non-financial support for the review, and the role of the funders or sponsors in the review.	Funding
Competing interests	26	Declare any competing interests of review authors.	Competing interests
Availability of data, code and other materials	27	Report which of the following are publicly available and where they can be found: template data collection forms; data extracted from included studies; data used for all analyses; analytic code; any other materials used in the review.	Data availability
